# Marriages of Consanguinity

**Published:** 1860-05

**Authors:** 


MARRIAGES OF CONSANGUINITY.—Since the publication in our journal of the able and interesting papers on marriages of consanguinity, by Doctor, now Professor, Bemiss, of Louisville, the subject has attracted much attention, both at home and abroad. Last winter the Governor of Kentucky called the attention of the Legislature of that State to it prominently, in his annual message, in the hope that they might be induced to enact some law for the purpose of preventing the formation of such alliances; and from the subjoined remarks, copied from a political journal, it would seem that at least one other State of the Union is interesting herself in a similar manner. If the statements contained in the extract be true, they are enough to make one shudder for the unfortunate offspring of such connections.

"The Ohio Legislature has been passing some laws on the subject of the intermarriage of near relatives. It is said that in Massachusetts, out of 17 families formed by the marriage of cousins, there were 95 children, and in Ohio, in 879 such families, there were 3900 children. It would thus seem that the average number of children is not diminished by such intermarriages, the Massachusetts statistics giving 5½ children to each such marriage. But out of these 95 children, 44 were idiots, 12 scrofulous, and only 37 in tolerable health; while in Ohio 2400 out of 3900 were either intellectually or physically defective. In one case of the marriage of double cousins, nine children, all there were, were idiots of low grade."

